# Improve sensitization and corrosion resistance of an Al-Mg alloy by optimization of grain boundaries

**DOI:** 10.1038/srep26870

**Published:** 2016-05-27

**Authors:** Jianfeng Yan, Nathan M. Heckman, Leonardo Velasco, Andrea M. Hodge

**Affiliations:** 1 Department of Aerospace and Mechanical Engineering, University of Southern California, Los Angeles, CA 90089, USA; 2 Department of Chemical Engineering and Materials Science, University of Southern California, Los Angeles, CA 90089, USA

## Abstract

The sensitization and subsequent intergranular corrosion of Al-5.3 wt.% Mg alloy has been shown to be an important factor in stress corrosion cracking of Al-Mg alloys. Understanding sensitization requires the review of grain boundary character on the precipitation process which can assist in developing and designing alloys with improved corrosion resistance. This study shows that the degree of precipitation in Al-Mg alloy is dependent on grain boundary misorientation angle, adjacent grain boundary planes and grain boundary types. The results show that the misorientation angle is the most important factor influencing precipitation in grain boundaries of the Al-Mg alloy. Low angle grain boundaries (≤15°) have better immunity to precipitation and grain boundary acid attack. High angle grain boundaries (>15°) are vulnerable to grain boundary acid attack. Grain boundaries with adjacent plane orientations near to {100} have potential for immunity to precipitation and grain boundary acid attack. This work shows that low Σ (Σ ≤ 29) coincident site lattice (CSL) grain boundaries have thinner β precipitates. Modified nitric acid mass loss test and polarization test demonstrated that the global corrosion resistance of sputtered Al-Mg alloy is enhanced. This may be attributed to the increased fractions of low Σ (Σ ≤ 29) CSL grain boundaries after sputtering.

Al-Mg 5xxx series alloys are commonly used in marine environments, such as naval ships, pressure vessels and aquatic hulls due to their excellent high strength, weldability and favorable corrosion resistance^1^. In contrast to heat treatable alloys such as the 2xxx, 6xxx, and 7xxx series, in which a desired level of mechanical strength is achieved by thermal heat treatments for precipitation hardening purposes, strength in Al-Mg 5xxx alloys is achieved primarily by solid solution strengthening, dispersion hardening, and/or work hardening^2^. However, a drawback for the use of Al-Mg 5xxx series alloys (Mg > 3 wt.%) is the susceptibility to sensitization and subsequent intergranular corrosion. During sensitization of Al-Mg alloys, Mg atoms preferentially diffuse to the grain boundary (GB) and form precipitation of β phase (Al_3_Mg_2_). The precipitation behavior of Al-Mg alloys has been the subject of many research studies, where the focus is to investigate the manner in which stable and metastable precipitating phases (β’ and β’’) nucleate and develop from a supersaturated solid solution^3^,^4^,^5^,^6^,^7^,^8^. It is generally suggested that β phase precipitation in binary Al-Mg alloys are formed following the reaction^3^,^4^,^5^,^6^,^7^,^8^:


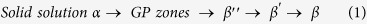


The intergranular β phase corrodes preferentially compared with the Al matrix in most environments, which leads to intergranular corrosion and stress corrosion cracking^9^,^10^,^11^.

Many studies have demonstrated that β phase precipitation and corrosion of Al alloys depend on the chemical composition^12^,^13^,^14^, mechanical processing^15^,^16^,^17^ , and elevated-temperature exposure^18^,^19^. For example, Zhang *et al.* studied the influence of grain size and grain orientation on the sensitization of Al 5083 processed by rolling, equal channel angular processing and high-pressure torsion^17^. The effect of aging time and temperature on the degree of sensitization of Al-Mg 5xxx series alloys was reported by Yi *et al.*^19^. Most recently, the effect of heat treatment temperature and time on the sensitization of Al 5083 was extensively reviewed^20^. However, there is still limited information about the effect of GB character on precipitation and the subsequent intergranular corrosion of Al-Mg alloys^21^,^22^. Usually, five variables are needed to describe a GB: one variable defines misorientation angle, two variables describe the misorientation axis, and the other two variables characterize GB plane orientation. Davenport *et al.* studied β phase intergranular precipitation of sensitized Al 5182 by etching samples with H_3_PO_4_ acid^23^. The results showed that the degree of precipitation and susceptibility to acid attack for a boundary were related to crystallographic misorientation. Low angle boundaries (<20°) were immune to acid attack due to the absence of β phase precipitation. In contrast, Kaigorodova *et al.* showed that precipitation existed at GBs with low angle misorientation (5–10°)^24^. Scotto D’Antuono *et al.* suggested that β precipitation was more prevalent along low-angle GBs than high-angle GBs, conflicting with previous observation of β phase growth only occurring in high angle GBs^21^. Zhao *et al.* demonstrated that some low angle GBs can have β precipitation while some high angle GBs are immune to β precipitation^22^, which is in agreement with the Scotto D’Antuono *et al.*^21^ , but in contrast to Davemport *et al.*^23^. In addition to GB misorientation angle, GB plane orientation can be important in determining the GB properties in polycrystalline materials as elucidated by Homer *et al.*^25^. These studies showed that further research is still needed to identify the effect of GB character on sensitization and corrosion behavior of Al-Mg alloys.

The coincident site lattice (CSL) model along with dislocation models presents a convenient way to describe the structure of GBs. The notation 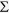 describes the degree of coincidence of lattice sites at a GB^26^. Since boundaries in polycrystalline specimens are usually not exact CSL boundaries, it is customary to divide boundaries into special GBs and general boundaries according to Brandon’s criterion^27^. The concept of “grain boundary design and control” has been introduced with the primary purpose of improving the bulk properties of polycrystalline materials through enhancement of the percentage of ‘special’ GBs^28^. Special GBs, which are low 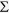 boundaries, have been shown to improve chemical and physical properties relative to general or high 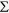 interfaces^29^,^30^,^31^. The work by Lin *et al.* showed that increasing the special GB frequency in Inconel Alloy 600 resulted in a proportional decrease in bulk intergranular corrosion susceptibility^32^. Zhou *et al.* found that more than 90% of special GBs (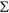 ≤ 29) exhibited immunity to sensitization for 304 L stainless steel^33^. The work by *Zhao et al.* demonstrated that the corrosion properties of Cu are improved by increasing the fraction of special 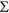
3 GBs^34^. These studies suggest that the corrosion properties of metals or alloys can be manipulated by adjusting the fraction of special GBs in the microstructure. However, the role of special GBs in the precipitation and corrosion properties in Al alloys is still not clear.

The present research is motivated by the practical viewpoint of obtaining materials with enhanced resistance to sensitization and intergranular corrosion, but also by the desire to extend our knowledge about the effect of GB character on the precipitation and immunity to acid attack for different GBs. Thus this manuscript will focus on studying the precipitation and acid etching behaviors of GBs with different character and the corrosion behavior of Al-Mg alloys. Scanning electron microscopy (SEM), transmission electron microscopy (TEM), electron backscatter diffraction (EBSD), and transmission electron backscatter diffraction (t-EBSD) techniques were used to analyse the relation between GB character and resistance to sensitization and subsequent intergranular corrosion properties. An expanded insight into how the boundary character correlates with precipitation formation and acid etching behaviors in Al-Mg alloys is presented.

## Result and discussion

### Effect of GB character on GB precipitation

#### Effect of grain misorientation

**Figure 1:**
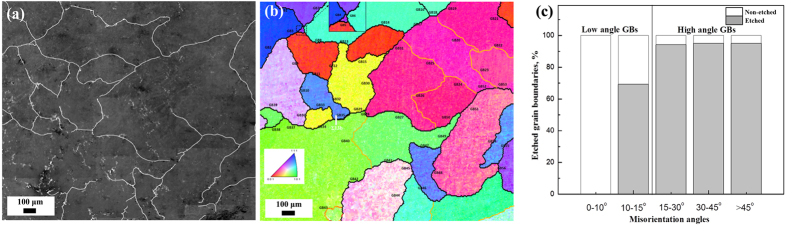
Top-surface SEM (**a**) corresponding EBSD grain orientation map (**b**) of sensitized received Al-Mg alloy after phosphoric acid etching. High-angle GBs (>15°) are denoted by the black lines, while low-angle GBs (≤15°) shown as yellow lines. The special GBs of 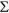
13b are marked by white rectangle; (**c**) Length percent of non-etched/etched GBs with different misorientation angles for sensitized received Al-Mg alloy after phosphoric acid etching.

The sensitization and subsequent corrosion properties of Al-5.3 wt.% Mg alloys are attributed to the formation of precipitates in the GBs. This section includes a discussion about the effects of GB character on precipitation by a phosphoric acid etching method. Figure 1a,b represent the top-view SEM topography for sensitized received Al-Mg alloy after etching by 10% phosphoric acid and corresponding EBSD orientation map of the same area. 56 GBs are numbered from GB1 to GB56 as shown in the EBSD orientation map (Fig. 1b). High-angle GBs (>15°) are denoted by the black lines, while low angle GBs (≤15°) are shown as yellow lines. A special 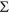
13b GB is marked by a white rectangle. Supplementary Table S1 shows the statistical analysis of the etching behaviors of 56 GBs with the different GB characters.

All 56 GBs are categorized into three types according to their etching behaviors: i) GBs that show continuous attack are called “fully-etched boundaries,” ii) GBs that show discontinuous attack are called “partially-etched boundaries,” and iii) GBs that show no attack are called “non-etched GBs”. The lengths of etched and non-etched regions for every GB were measured, and the length percent of non-etched/etched GBs at different misorientation angles are illustrated in Fig. 1c. When the misorientation angle is lower than 10°, all GBs show good immunity to acid attack. For the GBs with misorientation angle from 10° to 15°, the percent of etched length is 70%. The percent of etched GB length with high misorientation angle (>15°) is 95%. It is evident that the misorientation angle has a significant influence on the etching behaviors of GBs. It has been shown that more precipitates formed in high angle GBs compared with low angle GBs^23^. The results from this study indicate that indeed high angle GBs have worse immunity to acid attack, since the precipitates formed (Al_3_Mg_2_) are anodic to the aluminum matrix^9^.

The effect of GB misorientation angle on β precipitation can be explained by the difference in GB energy. The driving force for precipitation is proportional to the reduction in the Gibbs free energy, Δ*G*, and can be expressed as follows^35^:


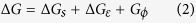


where Δ*G*_*s*_ is the surface free energy term, Δ*G*_*ε*_ is the strain energy term, and Δ*G*_*Φ*_ is the chemical free energy change.

It is expected that GBs with low energy will have higher activation energy for atom diffusion and it will be more difficult to form precipitates along low energy GBs. It has been observed that the GB energy and mobility of GBs increase as the misorientation angle increases from 2° to 15° and after this stage the GB energy is almost independent of the misorientation angle in Al alloys^36^,^37^,^38^. This is consistent with the precipitation and phosphoric acid etching results in this study, where low angle GBs with misorientation angle (≤15°) show lower vulnerability to acid attack and high angle GBs with large misorientation angle (>15°) are more vulnerable to acid attack.

While GB misorientation seems to be the most important factor in the formation of β precipitation, there are several exceptions in which misorientation angle does not predict the precipitation behavior. Overall, most of the high angle GBs are fully etched, which indicates high precipitate formation while most of the low angle GBs show immunity to precipitation and thus acid attack. However, there are also some GBs (such as GB9, GB17) with high misorientation angle (>15°) that are not etched or just partially etched and some GBs (GB24, GB43) with low misorientation angle (≤15°) that were vulnerable to acid attack. The special 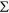
13b GB (GB35) showed immunity to acid attack although it has a high misorientation angle (27.8°). In previous studies, it was found that low angle GBs can have β precipitation and some high angle GBs are immune to β precipitation^21^,^22^, which is in agreement with our experimental results. Therefore, it can be suggested that the misorientation angle may not be the only parameter that affects the GB precipitation and etching behaviors.

#### Effect of GB plane orientations

**Figure 2:**
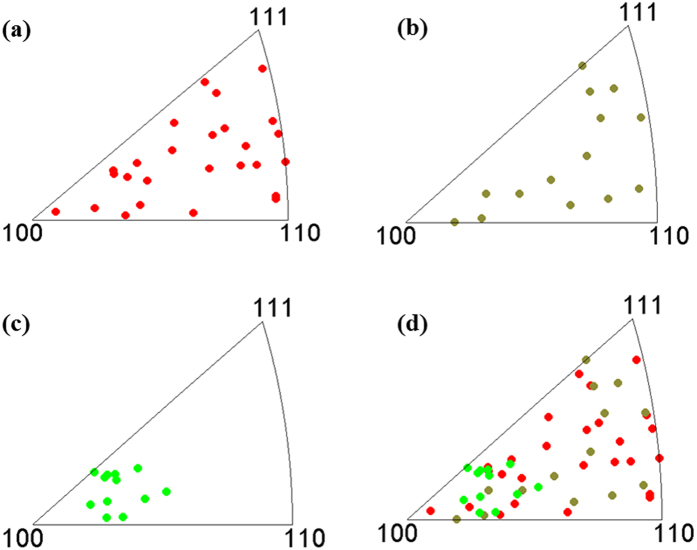
GB plane orientations of sensitized received Al-Mg alloy in standard triangles for (**a**) fully-etched boundaries, (**b**) partially-etched boundaries, (**c**) non-etched boundaries, (**d**) all boundaries.

Previous reports have shown that precipitation in GBs may be related to the plane orientations of the GBs^21^,^22^,^25^. The GB plane orientations were estimated by analyzing the top-surface EBSD orientation maps and the GB traces^22^,^39^. The effect of GB plane orientations on precipitation in GBs is considered based on the phosphoric acid etching method. Figure 2 presents the GB plane distribution in standard triangles for different boundaries in sensitized Al-Mg alloys. For fully-etched and partially-etched boundaries, the GB plane orientations are uniformly distributed in the standard triangles. It is interesting that plane orientations of non-etched boundaries are near to {100} orientations. This result suggests that the precipitation and etching behavior of GBs may depend not only on their misorientation angle, but also on the GB plane orientations. Previous studies have shown that GB orientation can play a role in β precipitation, and GBs with plane orientations near to {110} may facilitate β precipitation^22^. In this study, analysis of etching behaviors of 56 GBs reveals that the GBs with plane orientations near to {100} may have immunity to β precipitation and GB acid attack.

The different etching behaviors for GBs with plane orientations close to {100} may be attributed to the effect of plane orientation on the nucleation of β precipitation. It has been demonstrated that GB plane orientation has a significant effect on the nucleation of precipitates^40^. When the GB plane is close to the habit plane of a particular variant, copious nucleation in that particular variant occurs. These results suggest that the {100} plane orientation may be more resistant to acid attack because it is not the habit plane for nucleation of β precipitates.

#### Precipitation in special 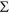 GBs

Coincident site lattice (CSL) boundaries are boundaries with special character. CSL boundaries are special because they have a given fraction of atoms in the GB plane which are coincident to both lattices separated by the GB. The 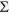 value denotes the fraction of atoms in coincidence. The CSL boundaries for the as-received sample were identified from orientation image microscopy (OIM) software data using Brandon’ criteria^27^:


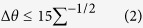


where Δθ is the angular deviation from the exact CSL and 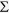 is the type of CSL boundary^41^.

**Figure 3:**
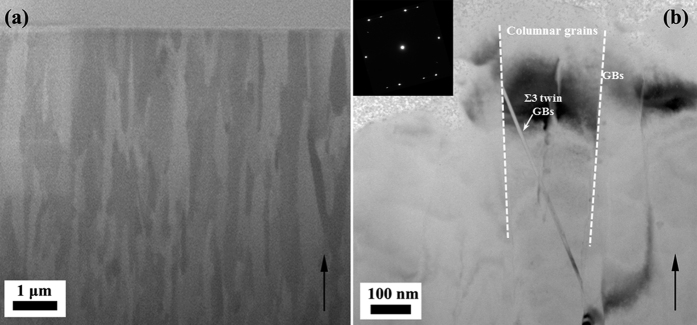
FIB and TEM cross-sectional micrographs of sputtered Al-Mg alloy (**a**) FIB cross-sectional image, Bright field TEM (**b**) showing columnar grains. Inset image shows SAED pattern.

**Figure 4:**
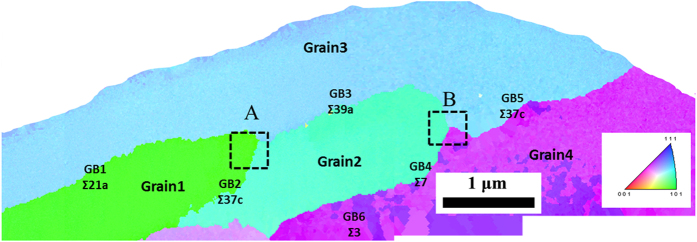
Cross sectional transmission electron backscatter diffraction image of sensitized sputtered Al-Mg alloy. The colour of the grain corresponds to the plane orientation.

**Figure 5:**
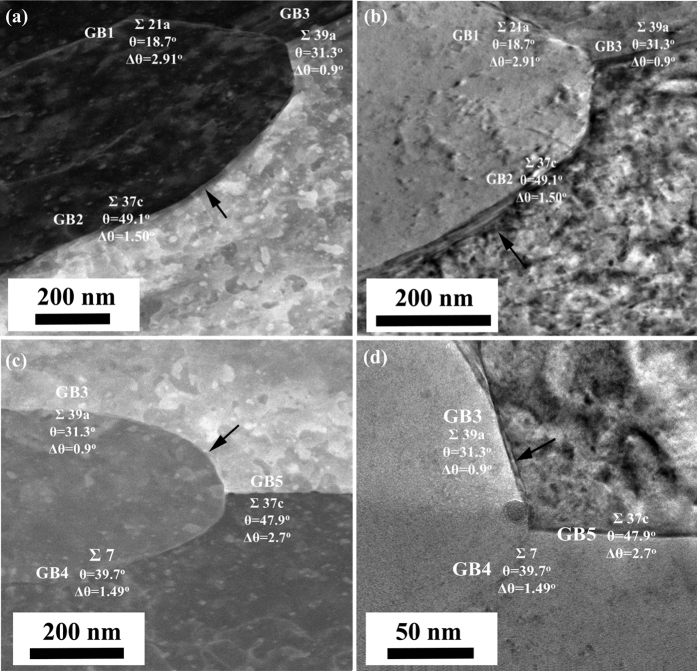
STEM image (**a**) and TEM image (**b**) (area A in Fig. 4); STEM image (**c**) and TEM image (**d**) (area B in Fig. 4) of sensitized sputtered Al-Mg alloy (Arrows indicate the precipitation at GBs).

It has been shown that GB35, marked by a white rectangle in Fig. 1b, is a special 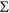
13b GB with misorientation angle of 27.8°. GB35 showed good immunity to GB acid attack. This indicates that the special GBs may have a role in enhancing the immunity to β precipitation and GB acid attack. In order to further explore the precipitation in special GBs, an Al-5.3 wt.% Mg alloy was sputtered. Microstructural analysis was performed on the as-sputtered and sensitized sputtered samples. FIB and TEM cross-sectional micrographs of sputtered Al-Mg alloy can be found in Fig. 3. The arrows indicate the {111} growth direction of the film during sputtering. In sputtered Al-Mg alloy, columnar grains with average grain size around 200 nm are observed. Figure 3b shows that some nanotwins (which contain 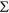
3 special GBs) exist in the columnar grains, which are reflected in the corresponding inset selected area electron diffraction (SAED) patterns. Since the grains in sputtered Al-Mg alloy are very fine, it is not effective to evaluate the precipitation in sensitized sputtered Al-Mg alloy by acid etching method using conventional EBSD and SEM screening. A combination of t-EBSD and STEM/TEM analysis is used to characterize the precipitation in sensitized sputtered Al-Mg alloy without the acid etching process. Figure 4 shows t-EBSD orientation maps of sensitized sputtered Al-Mg alloys, in which the colour of the grain corresponds to the plane orientation. The GB parameters 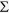, misorientation angle θ, and angular deviation Δθ are analysed with OIM software. In order to reveal the precipitation with different GBs, STEM and TEM were performed. Figure 5a,b show STEM and TEM images of area A in the t-EBSD image of Fig. 4. It is clear that the precipitate thickness varies at different GBs. GB1 is a 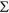
21a special GB with θ of 18.7°, GB2 is 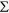
37c with θ of 49.1°, and GB 3 is 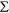
39a with θ of 31.3°. It is observed that the β precipitation at GB1 is much thinner than that at GB2 and GB3. This is expected since GB1 has a low angle GB misorientation. Figure 5c,d show the STEM and TEM images of area B in the t-EBSD image of Fig. 4. The precipitation at GB3 and GB5 is clearly observed, which is expected since they are both high angle GBs. Although GB4 has a high misorientation angle of 39.7°, the β precipitate is much thinner than the precipitates at other GBs. It is interesting to note that GB4 is a low 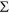
7 special GB. This suggests that less precipitation is formed at low 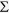 special GBs even if they have a high misorientation angle. Based on the t-EBSD image, there is also a special 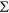
3 twin GB observed in the sensitized sputtered sample (GB6), and no precipitation could be detected by TEM analysis.

In order to visualize the precipitation at different special GBs, a plot of the precipitate thickness for different boundaries can be found in Supplementary Fig. S1. The average precipitation thickness at 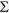
7 and 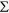
21a GBs is about 2 nm and 8 nm, respectively. The average precipitation thickness at 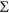
37c and 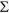
39a is higher, with values of 45 nm and 22 nm respectively. For special GBs the precipitation thickness seems to mainly depend on the 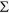 values, where low 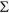 special GBs correlate with thinner β precipitation. This may be attributed to the low energy of low 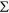 special GBs^42^,^43^. It is thought that the precipitation in GBs is mainly formed by the diffusion of Mg atoms in the Al matrix. The width of precipitation should be related with the diffusion coefficients and diffusion activation energy. As low 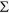 special boundaries have decreased energy, it is expected that the activation energy for atom diffusion will increase and form less precipitation. In general, it can be seen that less precipitation formed in low energy GBs such as low angle GBs or low 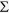 GBs^42^. Further studies are needed in order to explore the atom diffusion, nucleation, and growth process of precipitation in GBs with different GB energy.

### Corrosion Properties of different Al-Mg alloys

#### Modified nitric acid mass loss test

**Figure 6:**
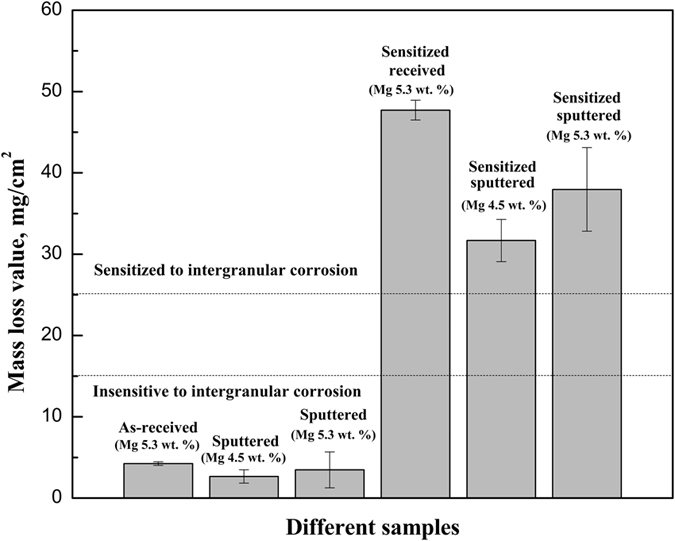
Mass loss value for different Al-Mg alloy samples in the modified NAMLT. Dotted lines are the classifications of degree of sensitization to intergranular corrosion: insensitive (<15 mg/cm^2^), indeterminate (15–25 mg/cm^2^), and sensitized (>25 mg/cm^2^).

The previous results in this manuscript demonstrated that the precipitation and immunity to acid attack of different GBs are related to their GB character including grain misorientation angle, plane orientations and GB type. Therefore, in order to provide a more comprehensive study, the global corrosion properties of different Al-Mg alloys including as-received, sensitized received, sputtered, and sensitized sputtered samples are further analysed. A modified nitric acid mass loss test (NAMLT) is used for quantitative measurement of susceptibility to intergranular corrosion of Al-Mg alloys^44^. To clarify the effect of Mg content on the mass loss values, samples with two different Mg contents of 4.5 wt.% and 5.3 wt.% were tested. Figure 6 shows mass loss value for different Al-Mg alloy samples in the modified NAMLT. Dotted lines are the classifications of degree of sensitization to intergranular corrosion. If the mass loss is higher than 25 mg/cm^2^, the sample is classified as sensitive to intergranular corrosion, whereas if the mass loss is below 15 mg/cm^2^, the sample is insensitive to intergranular corrosion^45^ . When the mass loss is between 15–25 mg/cm^2^ the sensitivity to intergranular corrosion is undetermined^45^. As shown in Fig. 6, the mass loss values for as-received and sputtered Al-Mg alloys are below 15 mg/cm^2^, which suggest that they are insensitive to intergranular corrosion. However mass loss values for both sensitized received and sensitized sputtered samples are higher than 25 mg/cm^2^, which show that they are sensitive to intergranular corrosion. The mass loss value is decreased for sensitized sputtered samples with lower Mg content, which suggests better intergranular corrosion resistance. It can be seen that the mass loss values for sputtered Al-Mg alloy samples are lower than that of as-received Al-Mg alloy samples, while the mass loss values for sensitized sputtered Al-Mg alloy samples are lower than that of sensitized received Al-Mg samples. These results suggest that the sputtered Al-Mg samples have better resistance to sensitization and intergranular corrosion.

The intergranular corrosion behavior of Al-Mg alloys is related to the diffusion of Mg to form intergranular β precipitation. As discussed previously, the immunity to precipitation and acid attack for different GBs seems to be affected by the GB character. In order to analyse how the boundary character interacts with intergranular corrosion properties for the different Al-Mg alloys, the GB character and distribution for both as-received and sputtered samples are determined by EBSD. Supplementary Fig. S2 shows an EBSD top-surface grain orientation map of as-received and sputtered Al-Mg alloys. The inset in the right top corner of the map is the top-surface grain orientation map. It is observed that plane orientations are randomly distributed for the as-received Al-Mg alloy sample while there is a significant {111} texture in the sputtered Al-Mg sample. As discussed previously, GBs with adjacent plane orientations near to {100} may have better immunity to β precipitation and acid attack. The sputtered sample has a columnar microstructure, and the GB plane orientation should be perpendicular to the {111} texture in the growth direction. Therefore, {100} planes cannot lie orthogonal to the {111} direction. It is not expected that there is a significant percentage of GB planes with a {100} direction in the sputtered samples.

The misorientation angle distributions of as-received and sputtered Al-Mg alloys are illustrated in Supplementary Fig. S3. It is shown that there is no increase of low angle grain boundaries for sputtered Al-Mg, which suggests that misorientation angle is not the reason for the improved corrosion resistance.

The fractions of low 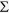 special GBs in as-received and sputtered Al-Mg alloy samples are analysed based on the EBSD maps and the results are summarized in Supplementary Table S2. It is clear that the fractions of low 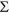 (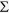 ≤ 29) special GBs in the sputtered Al-Mg alloy are increased compared with the as-received Al-Mg alloy. It has been shown in this study that the low 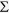 (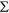 ≤ 29) special GBs usually have thinner β precipitation and better immunity to acid attack. Palumbo *et al.* used a geometric model to evaluate the potential effects of “special GB fraction” and average grain size on the intergranular stress corrosion crack susceptibility^31^ . It was shown that an improvement in intergranular stress corrosion resistance can be achieved by introducing a small fraction of corrosion resistant GBs^31^. These results indicate that the increased fractions of low 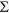 (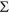 ≤ 29) special GBs may explain the better corrosion properties observed for sputtered Al-Mg alloy samples.

**Figure 7:**
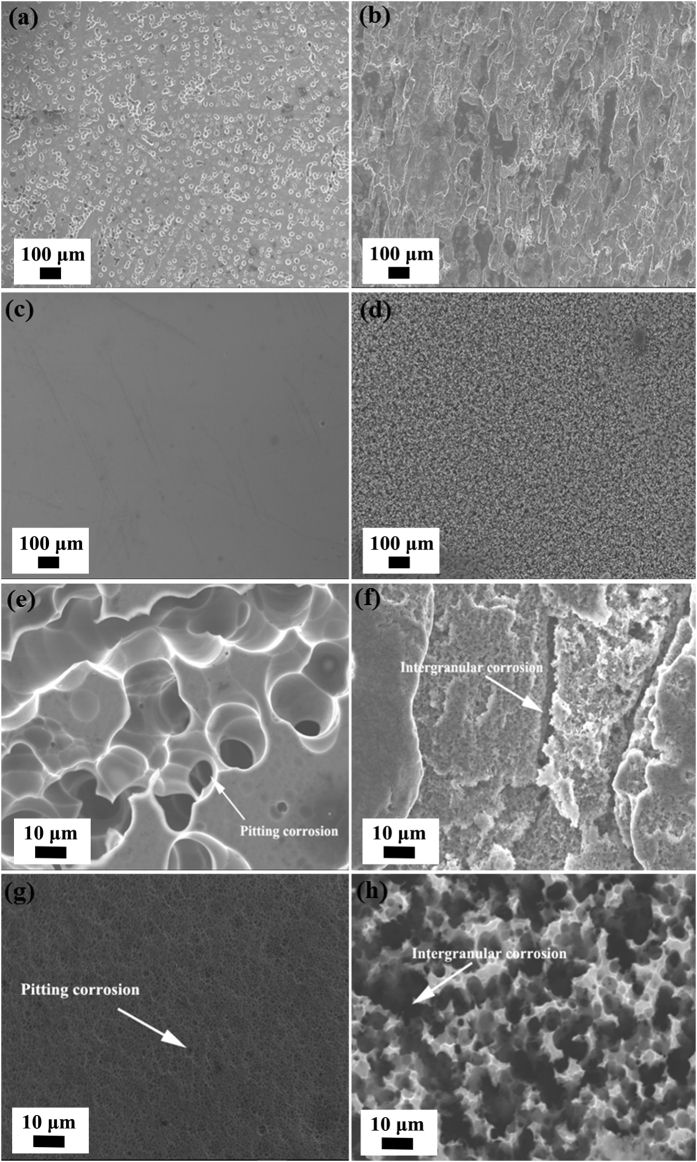
SEM top surface micrographs of Al-Mg alloy samples after modified NAMLT: low magnification of (**a**) as-received Al-Mg alloy; (**b**) sensitized received Al-Mg alloy; (**c**) sputtered Al-Mg alloy; (**d**) sensitized sputtered Al-Mg alloy; high magnification of (**e**) as-received Al-Mg alloy; (**f**) sensitized received Al-Mg alloy; (**g**) sputtered Al-Mg alloy; (**h**) sensitized sputtered Al-Mg alloy.

The typical corrosion morphology of different samples after modified NAMLT is shown in Fig. 7. For as-received (Fig. 7a,e) and sputtered samples (Fig. 7c,g), GB corrosion is characterized by isolated pitting which indicates that pitting corrosion is the main corrosion mechanism. The top surface of sputtered Al-Mg alloys (Fig. 7c,g) shows less pitting than the as-received (Fig. 7a,e) samples, which indicates better corrosion resistance. For sensitized received (Fig. 7b,f) and sensitized sputtered samples (Fig. 7d,h), the corrosion occurred preferentially along the GBs. This means that the intergranular corrosion is the main corrosion mechanism for the samples after sensitization. The intergranular corrosion in sensitized samples is a consequence of the formation of GB β precipitation. The precipitates in the GBs dissolves preferentially compared to the Al matrix which leads to the intergranular corrosion.

#### Polarization test

**Figure 8:**
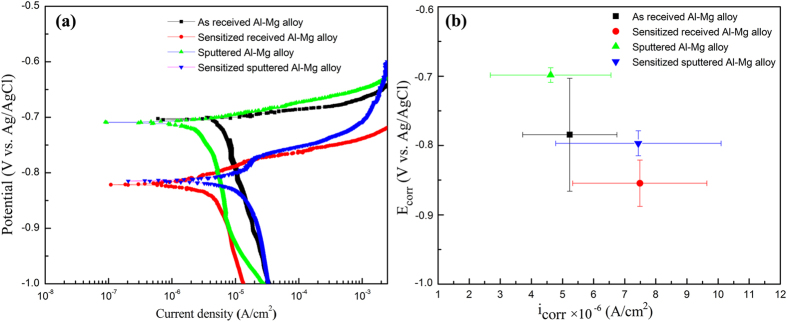
(**a**) Potentiodynamic polarization curves for different Al-Mg alloy samples in 3.5% NaCl solution with pH 3 at 22 °C obtained using a potential scan rate of 0.5 mV s^−1^; (**b**) The corrosion potential (E_corr_) as a function of current density values (i_corr_).

To further evaluate the corrosion properties of Al-Mg alloy samples, polarization tests were conducted. Fig. 8a shows typical potentiodynamic polarization behaviors observed for different Al-Mg alloy samples in the 3.5% NaCl solution. The Tafel extrapolation approach was used to extract the corrosion current density (i_corr_) and corrosion potential (E_corr_) from the polarization curves of Al-Mg alloy samples^46^ . It can be seen that the corrosion potential of sputtered Al-Mg alloy has a more noble value compared with that of as-received Al-Mg alloy. Similarly, the sensitized sputtered Al-Mg alloy has a higher corrosion potential value than that of sensitized received Al-Mg alloy, indicating a thermodynamic improvement in corrosion resistance. The overall corrosion potential for the sensitized samples has more negative values compared with samples without sensitization. This is due to the formation of β precipitation in the GBs, since the corrosion potential of the β phase is more negative than that of the Al matrix^9^ . The existence of β precipitation in the sensitized samples is confirmed by the EDS element mapping as shown in Supplementary Fig. S4. Large current density values reflect higher reaction rate between the specimen and corrosion agent. Compared with that of sensitized Al-Mg alloy, Al-Mg alloy without sensitization exhibited a lower current density, which indicates better resistance to corrosion. Figure 8b displays the change of E_corr_ as a function of i_corr_ to better illustrate these values for different Al-Mg alloys. The potentiodynamic polarization tests suggest that the sputtered samples are less susceptible to chloride ions corrosion, which is consistent with the above tests and discussions.

## Conclusions

The relationship between precipitation and GB character in Al-Mg alloys was comprehensively investigated. The precipitation of a total of 56 GBs in sensitized received Al-Mg alloy was evaluated by acid etching method using conventional EBSD and SEM methods. Grain misorientation was determined to be the most important factor affecting the precipitation and subsequent GB etching behavior. Low angle GBs (≤15°) had better immunity to precipitation and acid attack, whereas high angle GBs (>15°) were vulnerable to precipitation and acid attack. GB plane orientation could also play a role in the precipitation. A combination of t-EBSD and STEM/TEM analysis was used to characterize the precipitation in sensitized sputtered Al-Mg alloy. The results indicated that thinner precipitates usually formed at low 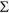 (≤29) special GBs which seemed to be related to their lower GB energy. Overall, these results showed that GB precipitation and immunity to acid attack depend on the GB character including misorientation angle, adjacent grain plane orientations and the 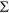 value for special GBs.

The global corrosion properties of Al-Mg alloy were tested by modified NAMLT and polarization tests and their GB character distribution were analysed. The results suggested that sputtered Al-Mg alloys had improved resistance to sensitization and intergranular corrosion. In modified NAMLT, mass loss values for sputtered Al-Mg alloy samples were lower than that of as-received Al-Mg alloy samples, while the mass loss values for sensitized sputtered Al-Mg alloy samples were lower than that of sensitized received Al-Mg samples. Compared with as-received Al-Mg alloys, sputtered samples showed lower mass loss and less pitting on the surface. In the polarization test, sputtered Al-Mg alloys had larger corrosion potential values and lower current density, which indicated a better corrosion resistance. The improved resistance to sensitization and intergranular corrosion of sputtered Al-Mg alloys may be attributed to the increased fraction of low 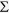 (≤29) CSL GBs.

## Methods

High purity Al-5.3 wt.% Mg alloys (purity 99.99%) were obtained from Plasmaterials, Inc., which are referred to as “as-received Al-Mg alloy” in this paper. “Sputtered Al-Mg alloy” was prepared by magnetron sputtering process using the as-received Al-Mg alloys as target materials. To sensitize the Al-Mg alloy samples, isothermal heat treatments were carried out at 175 °C for 7 days for both as-received and sputtered Al-Mg alloys. The samples after sensitization are referred to in this paper as “sensitized received Al-Mg alloy” and “sensitized sputtered Al-Mg alloy”.

Microstructural characteristics of as-received and sensitized received Al-Mg alloys were analysed by conventional electron backscatter diffraction (EBSD). The samples were mounted and ground with SiC paper, and then polished with diamond suspensions. The polisher (Buehler, Lake Bluff, IL) was used for the final polishing process with non-crystallizing colloidal silica suspension solution. The transmission electron backscatter diffraction (t-EBSD) technique was applied to test the grains in sensitized sputtered samples^47^. The specimen for t-EBSD and TEM analysis were prepared using focused ion beam (FIB) *in situ* lift-out technique^48^. The collated data with both EBSD and t-EBSD was analysed with OIM software. The TEM microstructural analysis was obtained using field emission transmission electron microscope (JEOL JEM-2100F) operating at 200 kV equipment with imaging detectors. TEM imaging was performed both in conventional transmission observation with parallel beam and in scanning transmission electron microscopy (STEM) mode with a probe size of 0.2 nm.

Phosphoric acid etching and subsequent screening test were used to evaluate the precipitation and immunity to acid attack for Al-Mg alloys. The samples were etched in 10 % H_3_PO_4_ at 35 °C for 1 min^22^,^39^. The GB character such as GB misorientation angle and plane orientation was obtained based on EBSD analysis. The corresponding intergranular corrosion behaviors for different GBs were checked using SEM (JEOL JSM-7001F). For corrosion tests, three samples from each type of Al-Mg alloy were tested using NAMLT^44^. The weight loss per unit of area before and after nitric acid immersion was calculated. Since the dimensions of the sputtered and received samples is different and the sputtered samples are foils, we used the terminology of “modified” NAMLT for the quantitative evaluation of susceptibility to intergranular corrosion for Al-Mg alloys^44^. Although, it has been shown that mass loss values are usually not affected by the sample dimension in NAMLT^45^. For electrochemical testing, at least two samples from each type of Al-Mg alloy were tested by using potentiodynamic polarization in a three-electrode cell filled with chloride solutions. The corrosion solution is artificial seawater, which is a naturally aerated 3.5% (35 g l^−1^) NaCl solution prepared by mixing ultrapure water with NaCl. HCl was added to the solution to adjust its pH value to 3. The temperature of the corrosion solution was kept at 23 ± 1 °C. The working electrode was the Al-Mg alloy sample, the reference electrode was silver/silver chloride (Ag/AgCl) and the counter electrode was a platinum wire. These three electrodes were connected to a Gamry Reference 3000 potenstiostat. The potentiodynamic polarization curves were analysed by Gamry Echem Analyst software.

## Additional Information

**How to cite this article**: Yan, J. *et al.* Improve sensitization and corrosion resistance of an Al-Mg alloy by optimization of grain boundaries. *Sci. Rep.* **6**, 26870; doi: 10.1038/srep26870 (2016).

## Supplementary Material

Supplementary Information
