# Supplementary material for: Improve sensitization and corrosion resistance of an Al-Mg alloy by optimization of grain boundaries
Source: Sci Rep. 2016 May 27;6:26870. doi: 10.1038/srep26870 (PMC4882518; doi:10.1038/srep26870)
Supplement: Supplementary Information [file srep26870-s1.pdf]

Supplementary Information

Improve sensitization and corrosion resistance of an Al-Mg alloy by optimization of grain boundaries

Jianfeng Yan[1], Nathan Heckman[1], Leonardo Velasco[2], Andrea M. Hodge[1,2*]

[1] Department of Aerospace and Mechanical Engineering, University of Southern California, Los Angeles, CA 90089, USA

[2] Department of Chemical Engineering and Materials Science, University of Southern California, Los Angeles, CA 90089, USA

* To whom correspondence should be addressed; E-mail: ahodge@usc.edu

Table S1

Summary of GBs with different parameters and the etching behaviors of sensitized received Al-Mg alloy. GB parameters were determined from EBSD results analysed with OIM. Etching behaviors were determined from SEM image.

ID	Mis. angle θ (°)	Misorientation axis	Grain orientations- G1	Grain orientation- G2	GB type	Etched GBs(mm)	Not etched GBs (mm)
GB1	33.8	<7 13 17>	7 -5 -2	-24 4 17	HA	0.485	0
GB2	44.3	<17 23 3>	-4 -1 6	22 15 0	HA	0.167	0
GB3	50.8	<-22 -1 20>	0 11 -13	-5 -1 29	HA	0.110	0
GB4	32.0	<-14 -4 -19>	2 16 -21	-2 8 -3	HA	0.065	0
GB5	42.4	<-13 -2 15>	-27 2 12	8 27 12	HA	0.062	0
GB6	42.0	<-17 23 5>	-5 -6 6	1 -8 -27	HA	0.033	0
GB7	47.9	<-9 -25 -14>	-18 -13 -2	9 -14 -20	HA	0.381	0
GB8	55.1	<22 11 -15>	14 -5 -1	13 15 10	HA	0.350	0
GB9	44.2	<-11 3 -9>	16 -25 -5	-3 9 1	HA	0.326	0.033
GB10	44.5	<19 -11 7>	13 -6 24	-12 -13 5	HA	0.330	0
GB11	48.4	<22 -17 4>	1 -23 -4	18 -12 -19	HA	0.187	0
GB12	36.1	<-8 7 -3>	-14 -2 25	20 9 -2	HA	0.316	0
GB13	58.6	<6 15 17>	19 -1 2	-5 3 -5	HA	0.100	0
GB14	52.7	<10 20 13>	2 23 1	-9 5 -2	HA	0.545	0
GB15	34.2	<-18 23 -8>	-3 -16 7	-20 23 0	HA	0.511	0
GB16	46.0	<13 -17 -15>	-21 8 2	-17 19 3	HA	0.233	0
GB17	53.8	<-17 -8 15>	19 -20 -6	3 9 22	HA	0.214	0.019
GB18	24.6	<5 1 -21>	-3 11 -24	2 -15 -25	HA	0.142	0
GB19	28.9	<25 -3 4>	27 1 5	7 0 -27	HA	0.458	0
GB20	5.5	<15 -9 -19>	21 -3 -7	5 -26 -8	LA	0	0.528
GB21	29.7	<5 -17 -25>	-27 6 5	17 -24 -1	HA	0.612	0

GB22	2.4	<-15 -11 21>	-3 -10 2	6 22 -5	LA	0	0.327
GB23	5.2	<23 -12 4>	-25 9 7	-4 16 -5	LA	0	0.309
GB24	10.7	<16 8 19>	8 1 2	9 -2 2	LA	0.327	0
GB25	5.3	<-13 19 -15>	-3 23 -5	5 20 2	LA	0	0.513
GB26	6.8	<11 -20 -20>	-6 8 -21	-3 8 18	LA	0	0.520
GB27	31.6	<-25 4 9>	27 -3 7	-28 4 -5	HA	0.607	0
GB28	31.1	<-11 -23 -7>	4 29 -4	27 -7 -11	HA	0.028	0.025
GB29	37.4	<-5 19 7>	16 -13 22	25 5 9	HA	0.133	0
GB30	29.7	<-6 -3 -1>	4 9 8	-20 6 -7	HA	0.546	0.033
GB31	17.1	<3 -5 0>	-1 30 0	-1 3 19	HA	0.202	0.028
GB32	54.4	<-15 19 2>	21 11 4	10 5 -9	HA	0.277	0.087
GB33	52.2	<-13 -16 2>	-5 -2 -6	13 -14 -19	HA	0.310	0
GB34	36.4	<-12 5 -7>	15 -6 -23	9 -3 10	HA	0.176	0.105
GB35	27.8	<19 -17 -16>	11 -14 -18	10 -11 -16	Σ13b	0.053	0.063
GB36	59.9	<-8 17 -19>	23 6 18	0 -5 -12	HA	0.138	0
GB37	45.0	<23 -14 6>	-13 21 9	8 4 5	HA	0.091	0
GB38	50.7	<-19 -22 5>	-6 -26 7	-2 -3 4	HA	0.100	0
GB39	32.4	<2 9 -8>	2 3 -13	1 -2 6	HA	0.124	0
GB40	6.5	<22 -14 15>	3 -30 -1	-18 3 -2	LA	0	0.566
GB41	25.2	<19 -2 11>	11 6 2	-9 -13 -18	HA	0.306	0.058
GB42	21.5	<9 4 13>	-1 -8 19	-19 -13 -4	HA	0.400	0
GB43	10.6	<-10 -27 -9>	5 13 -20	-14 11 24	LA	0.136	0.205
GB44	54.2	<6 -7 -2>	17 25 -3	-12 -11 17	HA	0.134	0.035
GB45	16.6	<15 12 -1>	7 21 2	-23 6 9	HA	0.094	0
GB46	47.1	<-3 21 -11>	13 -17 -21	-2 1 -2	HA	0.153	0
GB47	34.2	<2 -1 15>	12 -19 16	-21 11 -16	HA	0.423	0

GB48	44.7	<-13 21 11>	-8 6 21	15 10 1	HA	0.356	0
GB49	40.9	<-1 -8 -6>	-13 -20 13	11 25 3	HA	0.256	0.036
GB50	33.0	<-19 -4 3>	10 3 28	-5 19 -15	HA	0.166	0.075
GB51	34.9	<24 7 17>	0 -23 4	25 -9 -9	HA	0.207	0
GB52	37.5	<-27 14 4>	-4 3 -13	20 -13 -9	HA	0.117	0
GB53	29.5	<12 20 1>	-2 11 -6	-22 3 7	HA	0.167	0
GB54	45.8	<14 20 -17>	-28 7 6	-12 -9 17	HA	0.749	0.068
GB55	42.4	<-10 -5 2>	-3 -7 8	-23 4 -13	HA	0.426	0.041
GB56	37.4	<-8 -15 14>	8 -7 1	10 -3 -7	HA	0.057	0

Table S2

Length fraction of low Σ special GBs in as-received and sputtered Al-Mg alloy samples.

Boundary Type CSL(Σ)	Σ3	Σ5	Σ7	Σ9	Σ11	Σ13	Σ15	Σ17	Σ19	Σ21	Σ23	Σ25	Total
As-received Al-Mg alloy	0.015	0.005	0.009	0.004	0	0.021	0.003	0.002	0.005	0.010	0.001	0.009	0.084
Sputtered Al-Mg alloy	0.092	0	0.078	0.001	0.005	0.046	0.001	0.001	0.026	0.018	0	0.006	0.274

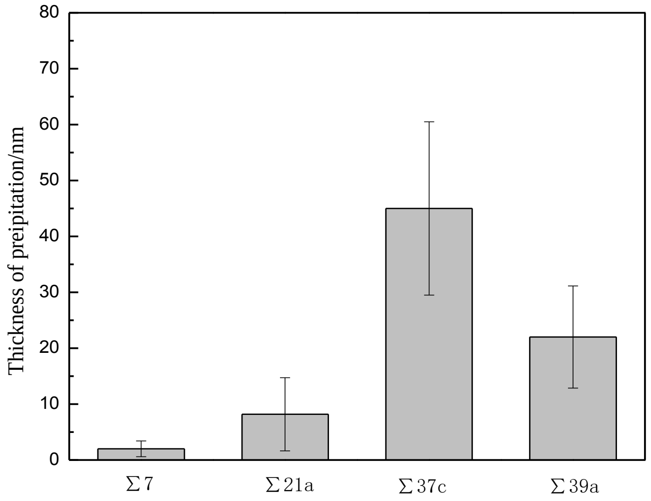

Figure S1. Precipitation thickness for GBs with different Σ values in sensitized sputtered Al-Mg alloy.

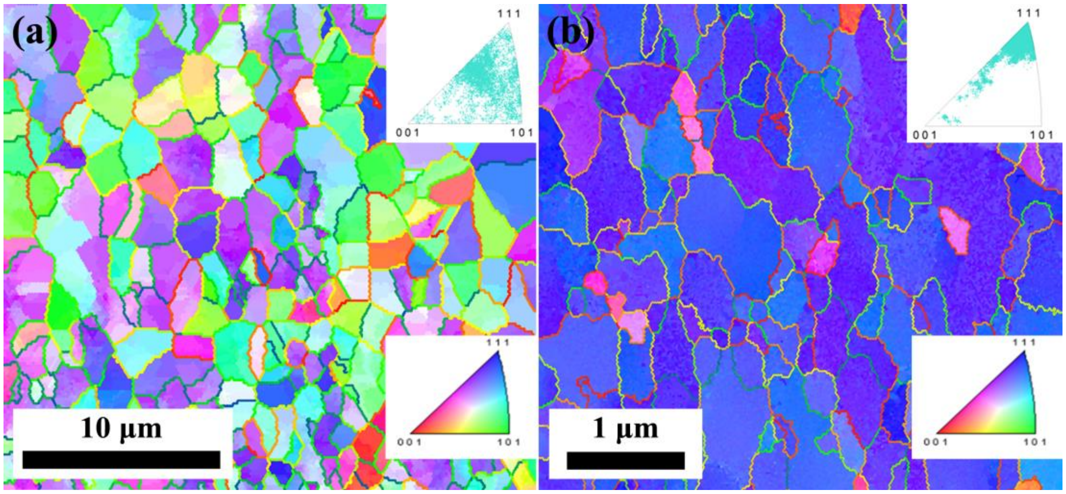

Figure S2. EBSD top-surface grain orientation map of as-received (a) and sputtered (b) Al-Mg alloys. Colours of GBs represent the misorientation angle, where green is the lowest (<15 °), yellow is ~30 °, and red is >45 °. The inset in the right top corner of the map is the top-surface grain orientation map, and the inset in the right bottom corner of the map is the triangle legend indicating the specific crystallographic orientations in the map. (Colour online)

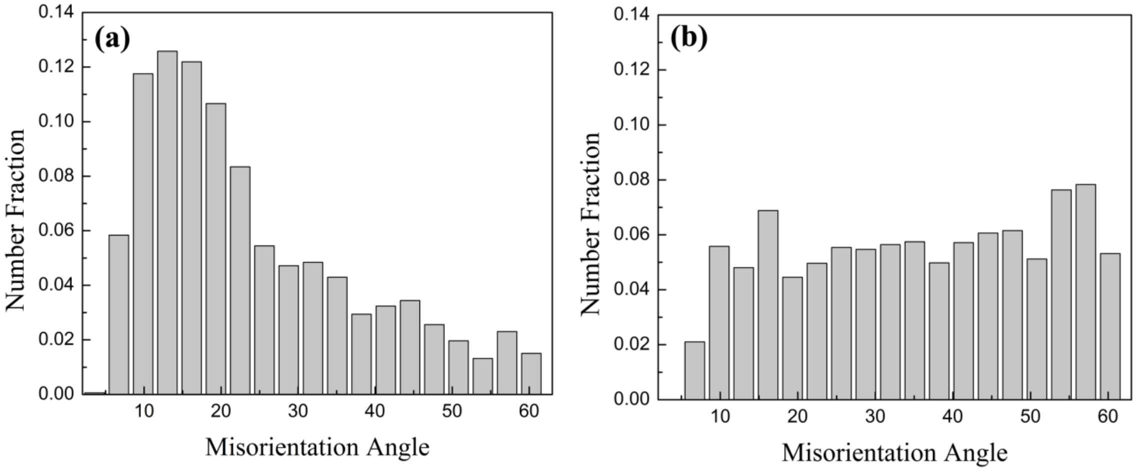

Figure S3. Misorientation angle distribution of Al-Mg alloys: (a) as-received Al-Mg alloy; (b) sputtered Al-Mg alloy.

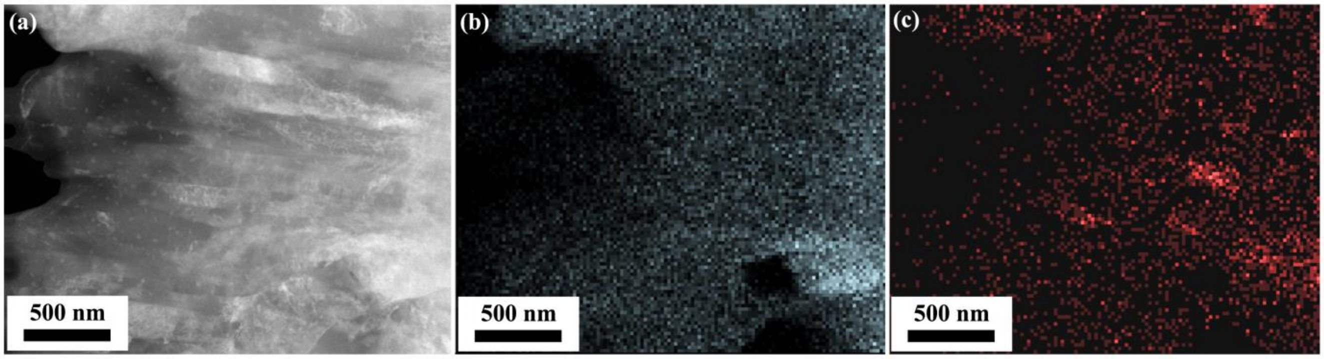

Figure S4. (a) STEM image and EDS maps (b and c) of sensitized sputtered Al-Mg alloy showing the distribution of Al and Mg, respectively. (Colour online)
